# Cell non-autonomous regulation of hepatic IGF-1 and neonatal growth by Kinase Suppressor of Ras 2 (KSR2)

**DOI:** 10.1038/srep32093

**Published:** 2016-08-26

**Authors:** Lili Guo, Diane L. Costanzo-Garvey, Deandra R. Smith, Megan E. Zavorka, Megan Venable-Kang, Richard G. MacDonald, Robert E. Lewis

**Affiliations:** 1 Eppley Institute for Cancer and Allied Diseases, University of Nebraska Medical Center, Omaha, NE 68198, USA; 2 Department of Biochemistry and Molecular Biology, University of Nebraska Medical Center, Omaha, NE 68198, USA; 3 Fred & Pamela Buffett Cancer Center, University of Nebraska Medical Center, Omaha, NE 68198, USA.

## Abstract

Individuals with poor postnatal growth are at risk for cardiovascular and metabolic problems as adults. Here we show that disruption of the molecular scaffold Kinase Suppressor of Ras 2 (KSR2) causes selective inhibition of hepatic GH signaling in neonatal mice with impaired expression of IGF-1 and IGFBP3. *ksr2*^−/−^ mice are normal size at birth but show a marked increase in FGF21 accompanied by reduced body mass, shortened body length, and reduced bone mineral density (BMD) and content (BMC) first evident during postnatal development. However, disrupting FGF21 in *ksr2*^−/−^ mice does not normalize mass, length, or bone density and content in *fgf21*^−/−^*ksr2*^−/−^ mice. Body length, BMC and BMD, but not body mass, are rescued by infection of two-day-old *ksr2*^−/−^ mice with a recombinant adenovirus encoding human IGF-1. Relative to wild-type mice, GH injections reveal a significant reduction in JAK2 and STAT5 phosphorylation in liver, but not in skeletal muscle, of *ksr2*^−/−^ mice. However, primary hepatocytes isolated from *ksr2*^−/−^ mice show no reduction in GH-stimulated STAT5 phosphorylation. These data indicate that KSR2 functions in a cell non-autonomous fashion to regulate GH-stimulated IGF-1 expression in the liver of neonatal mice, which plays a key role in the development of body length.

Low birth weight and neonatal growth defects are associated with obesity and the development of insulin resistance in adult humans^1^,^2^,^3^,^4^,^5^,^6^. Environmental factors like poor nutrition during fetal development and early life may activate mechanisms that impose a nutritionally thrifty phenotype upon individuals, promoting survival in the short term, but increasing susceptibility to type 2 diabetes (T2D) in the long term^1^,^3^. Various animal models have been developed to identify the molecular mechanisms that drive this metabolic reprogramming^7^,^8^. Most animal models require dietary, pharmacological, or surgical manipulation of the mother to limit nutrition or oxygen, which disrupts fetal development and suppresses the birth weight of pups. Modulating nutrition during the early neonatal period alters the development of central nervous system pathways critical to the development of normal adiposity and insulin sensitivity in adults^9^,^10^. In fact, the suckling period in rodents is critical for the development of pancreatic islets and the hypothalamus^8^,^11^. There is a paucity of genetic models, however, that reflect the effects of pre- or postnatal programming on the development of basal metabolism. Kinase suppressor of Ras 2 (KSR2) is required for normal neonatal growth as well as the development of normal energy balance and glucose homeostasis in the adult^12^,^13^. KSR2 knockout (*ksr2*^−/−^) mice may serve as a potential genetic model for assessing the importance of the neonatal period in the development of normal metabolism.

Kinase suppressor of Ras 1 and 2 (KSR1 and KSR2) are scaffold proteins for the Raf/MEK/ERK MAPK signaling pathway that mediates signaling downstream of cell-surface receptors^14^,^15^,^16^. KSR2, first discovered in *C. elegans*, is required for Ras-mediated signaling in germline meiotic progression, and functions redundantly with KSR1 in the development of the excretory system, hermaphrodite vulva, and male spicules^16^. Through an interaction with calcineurin, KSR2 mediates calcium-regulated activation of the ERK pathway. The KSR2 interaction with calcineurin is involved in neurite outgrowth^17^ and is required for optimal calcium uptake in white blood cells^18^. In addition to its role as a scaffold for ERK signaling, KSR2 also interacts with and regulates the energy sensor 5′-AMP-activated protein kinase (AMPK), an interaction that is a critical contributor to energy balance^12^. Humans with KSR2 mutations exhibit childhood hyperphagia, early-onset obesity, reduced metabolic rate, and severe insulin resistance^19^. *ksr2*^−/−^ mice recapitulate the key metabolic characteristics of humans with KSR2 mutations; *ksr2*^−/−^ mice are obese, insulin-resistant, and exhibit a reduced metabolic rate^12^. These observations suggest a conserved function of KSR2 between rodents and humans. The molecular mechanisms regulating KSR2-dependent insulin resistance and obesity are unknown.

In addition to obesity and severe insulin resistance in mature animals, *ksr2*^−/−^ mice grow more slowly than their wild type (WT) littermates during the neonatal period, yet they have a normal birth weight^12^. Postnatal growth is primarily regulated by growth hormone (GH)-induced insulin-like growth factor 1 (IGF-1)^20^. GH is secreted by the pituitary under the control of growth hormone-releasing hormone (GHRH) and somatostatin, which are both produced and secreted by the hypothalamus. GH induces IGF-1 expression and its secretion from peripheral tissues including the liver, muscle, fat, and bone. The main source of circulating IGF-1 is the liver. Circulating IGF-1, together with locally secreted IGF-1, promotes body growth^20^. In response to poor nutrition, the body induces GH resistance in order to decrease circulating IGF-1 and reduce growth and energy demand^21^. Recent studies suggest that increased fibroblast growth factor 21 (FGF21), a metabolic hormone primarily produced by liver, is an important factor in the regulation of liver-specific GH resistance in response to poor nutrition^22^.

We investigated the molecular basis for the neonatal growth defects in *ksr2*^−/−^ mice. Here we show that, although GH levels are normal in *ksr2*^−/−^ mice at postnatal day 17 (PN17), serum IGF-1 is decreased at both PN6 and PN17 in *ksr2*^−/−^ mice. Serum FGF21 and hepatic FGF21 mRNA levels are increased in neonatal *ksr2*^−/−^ mice compared to their WT littermates. However, FGF21 disruption fails to restore normal body weight to neonatal *ksr2*^−/−^ mice. Exogenous IGF-1 rescued bone development, but also failed to restore normal body weight to neonatal *ksr2*^−/−^ mice. GH-induced phosphorylation of hepatic STAT5b and JAK2 are decreased in *ksr2*^−/−^ neonates *in vivo,* but not in isolated hepatocytes, suggesting that the GH signaling is modulated by KSR2 via a cell non-autonomous mechanism. Postnatal growth and adult-onset insulin resistance may be affected by similar environmental and genomic influences^4^. Elucidating the mechanisms underlying growth defects in neonatal *ksr2*^−/−^ mice may help reveal mechanisms critical to the development of normal adult energy balance and glucose homeostasis.

## Results

### Neonatal *ksr2* ^−/−^ mice have growth defects and decreased IGF-1 levels

**Figure 1:**
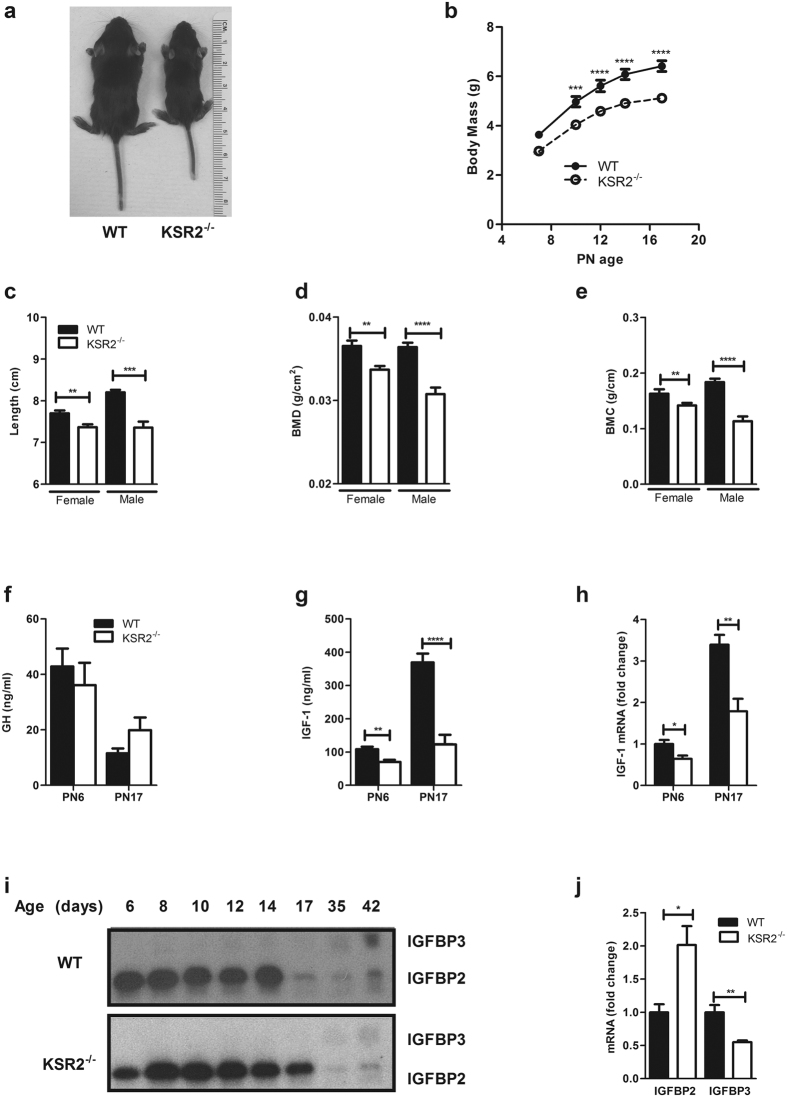
*ksr2*^−/−^ mice exhibit low serum IGF-1 and neonatal growth defects. (**a**) PN17 WT and *ksr2*^−/−^ mice littermates. (**b**) Growth curves of WT and *ksr2*^−/−^ mice from PN6 to PN17 (n = 11 WT group, n = 15 *ksr2*^−/−^ group, two-way ANOVA with repeated measures Bonferoni post hoc test). (**c–e**) Nose-to-anus length (**c**), bone mineral density (BMD) (**d**), and bone mineral content (BMC) (**e**) of WT and *ksr2*^−/−^ male and female mice at 5 weeks of age (n = 16, 15, 16, and 8, respectively). (**f**) Serum GH of WT and *ksr2*^−/−^ mice at PN6 (n = 7 per group) and PN17 (n = 9 per group). (**g**) Serum IGF-1 of WT and *ksr2*^−/−^ mice at PN6 and PN17. (n = 9, 7, 12, and 8, respectively). (**h**) Hepatic IGF-1 mRNA levels in WT and *ksr2*^−/−^ mice at PN6 and PN17 (n = 6–7 per group). For comparison, IGF-1 levels of WT mice at PN6 were set to 1 and rps18 was used as an internal control. (**i**) Analysis of serum IGFBPs from WT and *ksr*2^−/−^ mice. Aliquots (0.5 μl) of serum prepared from animals at the indicated postnatal times were subjected to ligand blot analysis probed with ^125^I-IGF2. The data shown are representative of duplicate runs for each of 3 distinct sets of serum samples. (**j**) IGFBP2 and IGFBP3 mRNA levels were measured by qPCR in PN17 liver tissue (n = 4–6 per group). Levels of WT mice were set to 1. Rps18 was used as an internal control. Data are shown as mean ± SEM. *p < 0.05, **p < 0.01, ***p < 0.001, ****p < 0.0001.

Though born normal in size, *ksr2*^−/−^ mice are only 80% and 70% the weight of WT littermates at PN6 and PN17, respectively (Fig. 1a,b). The growth defect is more severe in *ksr2*^−/−^ animals with the DBA/1LacJ background, which are about 50% the weight of their WT littermates at ages PN14-17 12. The nose-to-anus length is also decreased in *ksr2*^−/−^ mice compared to WT control mice at five weeks of age (Fig. 1c). Bone mineral density (BMD) and bone mineral content (BMC) are also significantly lower in *ksr2*^−/−^ mice relative to WT littermates at five weeks of age (Fig. 1d,e).

GH-induced IGF-1 is one of the major hormones regulating neonatal growth^20^. To determine if altered IGF-1 expression contributes to the neonatal growth and bone defects in *ksr2*^−/−^ mice, serum GH and IGF-1 levels in PN6 and PN17 WT and *ksr2*^−/−^ mice were measured by ELISA. Compared to WT offspring, *ksr2*^−/−^ mice show no significant differences in GH levels at both PN6 and PN17 (Fig. 1f). However, serum IGF-1 is 35% and 65% lower in *ksr2*^−/−^ mice compared to WT littermates at PN6 and PN17, respectively (Fig. 1g). As measured by qPCR, IGF-1 mRNA is decreased by 36% and 43% in the liver of *ksr2*^−/−^ mice at PN6 and PN17, respectively (Fig. 1h). Expression of serum IGF binding proteins (IGFBPs) was also measured. As observed previously by ligand blotting, the major IGFBPs expressed in the serum of neonatal mice are IGFBP2 and IGFBP3 23. Whereas IGFBP2 expression dominates in the early postnatal period, its serum concentration declines after PN14 in WT mice (Fig. 1i, top panel). However, in the *ksr2*^−/−^ mice, this decrease is delayed by several days and does not occur until after PN17 (Fig. 1i, bottom panel). IGFBP3 becomes the dominant serum IGFBP in adult mice, and serum IGFBP3 is low in *ksr*2^−/−^ mice as compared to WT mice at 6 weeks of age. Compared to WT mice, liver IGFBP2 mRNA levels are up-regulated, and IGFBP3 mRNA levels are down-regulated in *ksr2*^−/−^ mice at PN17 (Fig. 1j). Elevated IGFBP2 and decreased IGFBP3 are consistent with the reduction in GH signaling observed in *ksr2*^−/−^ mice^24^. These results show that KSR2 is required for GH-induced hepatic IGF-1 production and regulation of the IGF-1/IGFBP axis in neonatal mice.

### FGF21 is elevated in neonatal *ksr2* ^−/−^ mice

**Figure 2:**
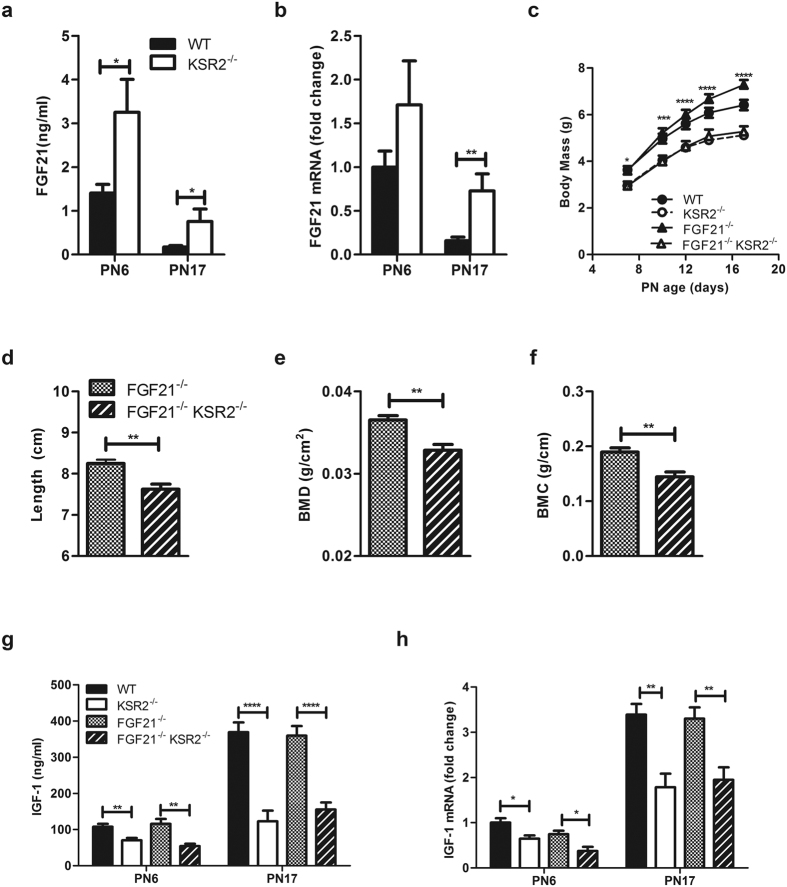
FGF21 disruption does not rescue the neonatal growth defect of *ksr2*^−/−^ mice. (**a**) Serum FGF21 levels in PN6 and PN17 WT and *ksr2*^−/−^ littermates (n = 6–9 per group). (**b**) Hepatic FGF21 mRNA in PN6 and PN17 WT and *ksr2*^−/−^ littermates (n = 6, 6, 10, and 9 respectively). Levels of WT mice at PN6 were set to 1 and rps18 was used as an internal control. (**c**) Growth curves of PN6 to PN17 WT, *ksr2*^−/−^, *fgf21*^−/−^, and *fgf21*^−/−^ *ksr2*^−/−^ male and female mice (n = 10–15 per group, two- way ANOVA with repeated measures Bonferoni post hoc test was used). (**d–f**) Nose-to-anus length (**d**), bone mineral density (**e**), and bone mineral content (**f**) of male *fgf21*^−/−^ and *fgf21*^−/−^ *ksr2*^−/−^ mice at 5 weeks of age (n = 8 per group). (**g**) Serum IGF-1 levels from male and female mice of the indicated genotypes at PN6 (n = 9, 7, 5, and 7 respectively) and PN17 (n = 12, 8, 9, and 12, respectively). (**h**) Hepatic IGF-1 mRNA levels from male and female mice of the indicated genotypes at PN6 (n = 4–6 per group) and PN17 (n = 7 per group). For comparison, IGF-1 levels of WT at PN6 were set to 1 and rps18 was used as an internal control. Data are shown as mean ± SEM. *p < 0.05, **p < 0.01, ***p < 0.001, ****p < 0.0001

FGF21 induces liver Suppressor of Cytokine Signaling 2 (SOCS2) overexpression to inhibit GH receptor signaling, subsequently decreasing IGF-1 mRNA expression in hepatocytes^22^,^25^. High levels of FGF21 have also been reported to cause bone loss by potentiating PPARγ activity^26^. Importantly, FGF21 transgenic mice have normal birth weights, but they exhibit stunted neonatal growth and decreased blood IGF-1 levels^22^, similar phenotypic characteristics to those observed in *ksr2*^−/−^ mice. Relative to WT mice, FGF21 is increased 2.4- and 4.4-fold in the sera of PN6 and PN17 *ksr2*^−/−^ mice, respectively (Fig. 2a). Hepatic FGF21 mRNA levels are slightly, but not significantly, elevated in *ksr2*^−/−^ mice at PN6. However, in PN17 *ksr2*^−/−^ mice, the levels of FGF21 are elevated 4-fold above WT levels (Fig. 2b). It is important to note that FGF21 levels in both *ksr2*^−/−^ and WT mice at PN17 are significantly lower compared to those at PN6 (Fig. 2a,b). This decline of FGF21 mRNA expression in the liver and of FGF21 protein levels in the sera of mice from ages PN6 to PN17 is consistent with a previous report^27^.

### FGF21 disruption does not rescue the neonatal growth defect of *ksr2* ^−/−^ mice

To evaluate if elevated FGF21 levels cause the growth defects and GH resistance in *ksr2*^−/−^ mice, *fgf21*^−/−^ mice (a gift from Dr. Steven A. Kliewer, UT Southwestern) were crossed with *ksr2*^+/−^ mice to produce the *fgf21*^−/−^ *ksr2*^−/−^ mice. *fgf21*^−/−^ *ksr2*^−/−^ mice are the same size and grow at the same rate as *ksr2*^−/−^ mice (Fig. 2c). At 5 weeks of age, nose-to-anus length, BMD, and BMC of *fgf21*^−/−^ *ksr2*^−/−^ mice are significantly less than their *fgf21*^−/−^ littermates (Fig. 2d–f), and are consistent with levels seen in *ksr2*^−/−^ mice (Fig. 1c–e). There are no detectable differences in serum IGF-1 (Fig. 2g) or hepatic IGF-1 mRNA (Fig. 2h) between *fgf21*^−/−^ *ksr2*^−/−^ mice and *ksr2*^−/−^ mice at both PN6 and PN17. This suggests that, although elevated blood FGF21 can inhibit IGF-1 expression, removing FGF21 does not increase IGF-1 levels in *ksr2*^−/−^ mice. In addition, the serum IGF-1 levels in *fgf21*^−/−^ mice are comparable with the levels of WT mice (Figs 1g and 2g) and consistent with previous reports^28^. These results suggest that elevated serum FGF21 is not the source of the growth and bone defects in *ksr2*^−/−^ neonatal mice.

### Exogenous IGF-1 restores body length and bone development, but not body mass in *ksr2* ^−/−^ pups

**Figure 3:**
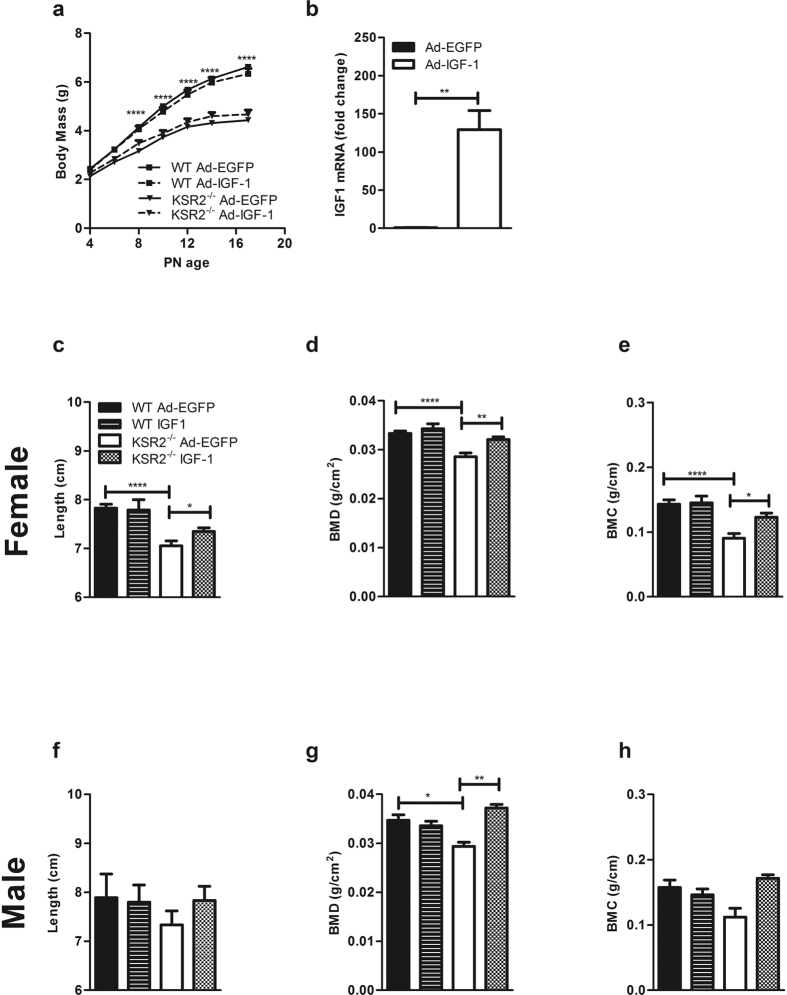
Exogenous IGF-1 rescues the defects in length and bone development, but not the mass of *ksr2*^−/−^ mice. (**a**) Growth curves of WT and *ksr2*^−/−^ injected with AdRSVeGFP (Ad-EGFP) or AdRSVhIGF-1 (Ad-IGF-1) adenovirus at PN2 (n = 16–21 per group, two-way ANOVA with repeated measures Bonferoni post hoc test was used). (**b**) Total IGF-1 (mouse and human) mRNA expression in PN5 *ksr2*^+/−^ liver (n = 4–6 per group). For comparison, the average IGF-1 mRNA level of Ad-EGFP-injected PN5 mice was set to 1. Rps18 was used as an internal control. (**c**–**e**). Nose-to-anus length (**c**), bone mineral density (**d**), and bone mineral content (**e**) at 5 weeks of age of WT and *ksr2*^−/−^ female mice injected with control or IGF-1-encoding adenovirus (n = 17, 7, 8, and 10 respectively). (**f–h**) Nose-to-anus length (**f**), bone mineral density (**g**), and bone mineral content (**h**) at 5 weeks of age of WT and *ksr2*^−/−^ male mice injected with control or IGF-1-encoding adenovirus (n = 10, 10, 3, 3, respectively). One-way ANOVA with Bonferroni post-test with multiple comparisons was used to analyze the data in panels c-h. Data are shown as mean ± SEM. *p < 0.05, **p < 0.01, ***p < 0.001, ****p < 0.0001.

To determine if low IGF-1 levels are responsible for the growth defects observed in *ksr2*^−/−^ mice, we expressed IGF-1 *in vivo* by injecting adenovirus expressing human IGF-1 (Ad5RSV-IGF-1) in newborn pups (PN2) by retro-orbital injection^29^. Compared to the control-injected (Ad5RSV-eGFP) *ksr2*^−/−^ mice, the Ad5RSV-IGF-1 injection failed to rescue the body mass of *ksr2*^−/−^ mice during the neonatal period (Fig. 3a). We measured liver IGF-1 mRNA expression in *ksr2*^+/−^ mice to confirm the expression of human IGF-1 (hIGF-1) via adenovirus. When measured 3 days after adenovirus injection (PN5), total IGF-1 mRNA (including both mouse endogenous mRNA and virally expressed human IGF-1) in mice injected with Ad5RSV-IGF-1 was markedly increased as compared to mice injected with the Ad5RSV-eGFP (Fig. 3b). Although body mass is not improved by adenovirus-mediated increased IGF-1 expression, at 5 weeks of age, Ad5RSV-IGF-1 normalizes nose-to-anus length, BMC, and BMD in female (Fig. 3c–e) and BMD in male ksr2^−/−^ mice (Fig. 3g–i) while having no significant effect on WT mice. These data are consistent with observations that serum IGF-1 is important for bone growth and bone density^30^,^31^. Our results suggest that serum IGF-1 contributes to neonatal skeleton development, but is not the only factor resulting in the decreased whole-body mass in *ksr2*^−/−^ mice.

### A cell non-autonomous action of KSR2 promotes GH signaling in the liver

**Figure 4:**
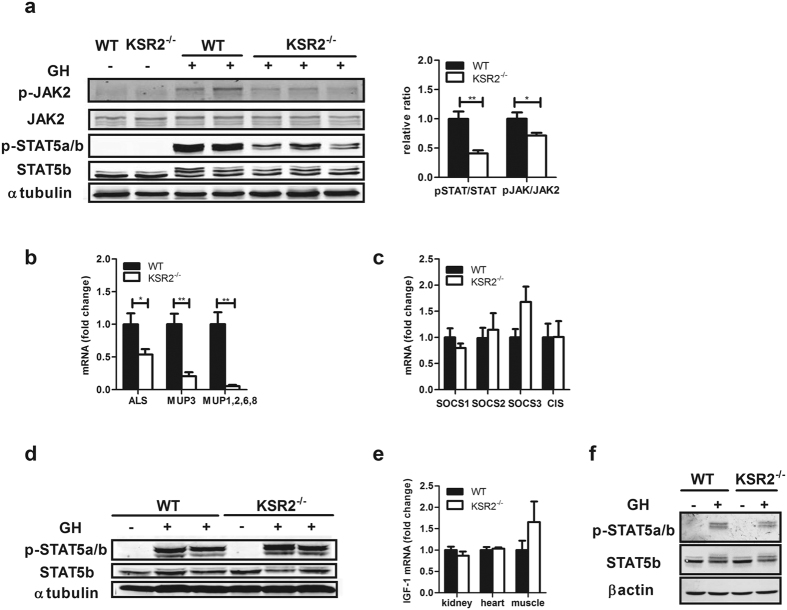
A cell non-autonomous action of KSR2 is required for GH signaling in the liver. (**a**) Hepatic STAT5b and JAK2 phosphorylation in PN17 WT and *ksr2*^−/−^ mice 15 min after IP injection of PBS or 125 μg/kg recombinant human GH. Liver tissue was lysed and analyzed via western blot with the indicated antibodies. The ratio of phosphorylation to total STAT5 and JAK2 was analyzed by LI-COR Odyssey system (n = 4–5 per group). Quantification is shown to the right of the western blot. (**b**) STAT5 target genes ALS, MUP3, and MUP1/2/6/8 were measured in livers from PN17 WT and *ksr2*^−/−^ mice (n = 4–6 per group). WT levels were set to 1. *p < 0.05, **p < 0.01. (**c**) qPCR of SOCS1-3 and CIS mRNAs from WT and *ksr2*^−/−^ liver (n = 6–8 per group). For comparison, levels of WT were set to 1 and rps18 was used as an internal control. (**d**) Muscle STAT5b phosphorylation in PN17 WT and *ksr*2^−/−^ mice treated as in panel a. (**e**) IGF-1 mRNA levels from kidney, heart, and muscle of PN17 WT and *ksr2*^−/−^ mice (n = 3–4 per group). IGF-1 levels of WT were set to 1, and 18srRNA was used as an internal control. (**f**) STAT5 phosphorylation in primary hepatocytes from PN17 WT and *ksr2*^−/−^ liver treated for 5 min with 100 ng/ml GH. Results are representative of three independent experiments. Data are shown as mean ± SEM. *p < 0.05, **p < 0.01.

To explore the role of KSR2 in GH-stimulated IGF-1 expression during the neonatal period, we studied GH signaling in *ksr2*^−/−^ hepatocytes. GH binding induces a conformational change in dimerized growth hormone receptor (GHR) leading to the transactivation of JAK2^32^,^33^ and phosphorylation of STAT5b, which is the major transcription factor driving GH-stimulated IGF-1 gene expression^34^. To understand the molecular mechanisms behind the decreased IGF-1 expression in the livers of neonatal *ksr2*^−/−^ mice, we injected GH intraperitoneally into PN17 WT and *ksr2*^−/−^ mice, euthanized the mice after 15 min, and prepared liver tissue for western blot analysis. Phosphorylation of both JAK2 and STAT5 is increased in the livers of WT mice in response to GH injection. However, in *ksr2*^−/−^ mice, GH-stimulated phosphorylation of JAK2 and STAT5 is substantially attenuated compared to their WT littermate controls (Fig. 4a). To further establish that STAT5b activity is decreased in neonatal *ksr2*^−/−^ mice, mRNA levels for the STAT5b gene targets ALS, MUP3, and MUP1/2/6/8 were measured by qPCR and shown to be down-regulated in *ksr2*^−/−^ mice compared to their WT littermates (Fig. 4b). These results suggest that the decreased blood IGF-1 levels in *ksr2*^−/−^ mice may be caused by suppressed GH signaling as represented by decreased JAK2 and STAT5b activities. However, the impaired GH signaling is not due to elevated expression of SOCS, as the *ksr2*^−/−^ liver expresses normal amounts of SOCS1-3 and the related Cytokine-Inducible SH2-containing (CIS) mRNA (Fig. 4c). To determine the extent to which KSR2 disruption affects GH signaling throughout the body, we also examined STAT5 phosphorylation in quadriceps muscle. GH stimulation induced similar STAT5 phosphorylation in WT and *ksr2*^−/−^ mice (Fig. 4d). Furthermore, IGF-1 mRNA levels from skeletal muscle, heart, and kidney in WT and *ksr2*^−/−^ mice at PN17 were not different (Fig. 4e). These data suggest that the GH resistance is specific to the liver.

To further understand how KSR2 regulates GH signaling specifically in the liver, primary hepatocytes were isolated from PN17 WT and *ksr2*^−/−^ livers and treated with 100 ng/ml of GH, and the cells were lysed. No difference was observed in STAT5b phosphorylation between WT and *ksr2*^−/−^ hepatocytes in response to GH stimulation (Fig. 4f), suggesting that KSR2 acts in a cell non-autonomous manner to regulate liver GH signaling.

## Discussion

The molecular mechanisms that underlie postnatal development have been studied extensively, but remain incompletely understood. As an extension of fetal development in mice, the postnatal period is important in initiating developmental plasticity^11^. Our data indicate that KSR2 functions in a cell non-autonomous fashion to promote postnatal signaling that optimizes growth hormone (GH)-stimulated hepatic IGF-1 expression. The reduced expression of IGF-1 in *ksr2*^−/−^ neonatal mice appears to account for defects in bone development and body length in these mice, but does not account for their reduced body mass. The developmental growth defect displayed by neonatal *ksr2*^−/−^ mice during the first three weeks of life is followed by accelerated weight gain that leads to obesity and insulin resistance in adult mice^12^,^13^. The effect of KSR2 on these developmental changes raises the possibility that the function of this molecular scaffold during the postnatal period affects adult metabolism.

Despite the fact that *ksr2*^−/−^ mice are born normal in size, the effects of KSR2 disruption show a striking similarity to the effects of intrauterine growth retardation (IUGR) on the IGF-1/IGFBP axis. IUGR is associated with a state of GH resistance, which is characterized by increased GH and reduced IGF-1 concentrations^35^. IUGR models show decreased IGF-1 from day 22 of gestation to postnatal day 9, but IGF-1 levels were not different at later time points^36^. Similarly, *ksr2*^−/−^ mice have low IGF-1 levels from PN6 to PN17 (Fig. 1), yet do not show significant differences in IGF-1 expression in adulthood (data not shown). Non-catch-up growth (NCG) in children born small for gestational age (SGA) is associated with GH resistance^37^,^38^,^39^,^40^. Similar to the effect of KSR2 disruption in mouse pups (Fig. 4), uterine artery ligation in pregnant rats to induce NCG-SGA also impaired GH-stimulated JAK2 and STAT5 phosphorylation^39^. IUGR children have lower serum IGFBP3 than lean children of normal height^41^, while postnatal *ksr2*^−/−^ mice also show decreased IGFBP3 (Fig. 3). These defects in GH signaling and IGF-1 and IGFBP expression suggest that KSR2 deletion disrupts a physiological pathway also impaired by IUGR. However, the fact that *ksr2*^−/−^ mice develop normally *in utero* suggests that the small size in rodent models of IUGR may not be the only trigger for altered postnatal programming of the GH/IGF-1 axis.

Rescue of growth in IUGR infants has been attempted by treatment with IGF-1 without consistent conclusions. Maternal administration of IGF-1 during the second half of pregnancy improves maternal weight, but not fetal and placental weights^42^,^43^. Transamniotic administration of recombinant hIGF-1 increased serum IGF-1 levels, but failed to significantly increase fetus weight^44^. However, fetal birth weight was increased by intraplacental injection of an Ad-IGF-1 gene in an IUGR rabbit model^45^. Our studies show that body mass cannot be rescued in *ksr2*^−/−^ mice using Ad-IGF-1 during the neonatal period, though bone length, BMC, and BMD were restored to normal. These data suggest that additional KSR2-dependent factors contribute to normal neonatal development.

Nutritional and metabolic status regulates the GH/IGF-1 axis through the liver by modulating liver sensitivity to GH^46^. FGF21, a member of the endocrine FGF superfamily, regulates growth and causes GH resistance under nutrient-deprived conditions. FGF21 is considered a starvation signal that mediates adaptive responses based on nutritional status^22^,^28^. FGF21 transgenic mice have a normal birth weight, but are significantly smaller than WT mice at three weeks of age^22^. *ksr2*^−/−^ mice showed higher FGF21 expression during the neonatal period (Fig. 2). Collectively, the growth similarities between neonatal *ksr2*^−/−^ mice and neonatal FGF21 transgenic mice suggested that the elevated expression of FGF21 could underlie the GH resistance in *ksr2*^−/−^ mice. However, the *fgf21*^−/−^*ksr2*^−/−^ mice are still smaller than WT mice and maintain a growth rate similar to *ksr2*^−/−^ mice. FGF21-induced GH resistance is mediated partly by SOCS2 overexpression^22^. However, *ksr2*^−/−^ mice have normal liver levels of SOCS and CIS. These observations, along with suppressed IGF-1 expression in the *fgf21*^−/−^ *ksr2*^−/−^ mice, demonstrate that elevated FGF21 is not responsible for defective GH signaling in *ksr2*^−/−^ mice.

The mechanism by which FGF21 is increased in *ksr2*^−/−^ neonatal mice is unknown. However, its acute elevation may offer clues regarding the stress caused by disruption of KSR2 in neonates. The function of neonatal FGF21 may be different from adult FGF21, as fasting cannot induce increased FGF21 expression in unfed or undernourished newborn pups as it does in adult mice^27^. Instead, the increase in neonatal FGF21 is induced by a high-fat diet and by milk intake. The increased FGF21 may suggest that neonatal *ksr2*^−/−^ mice have normal milk intake, which is consistent with the detection of milk in the stomachs of PN6 and PN17 *ksr2*^−/−^ mice when euthanized. Neonatal FGF21 is considered to be a food intake-dependent signal that favors neonatal brown adipose tissue thermogenic activation. *ksr2*^−/−^ mice have defective thermogenesis^12^, and the increased FGF21 may reflect a compensatory attempt to increase *ksr2*^−/−^ thermogenesis and promote survival. Human preterm infants have high FGF21 expression that is inversely related to infant height^47^. These observations suggest that, though elevated FGF21 does not affect neonatal growth in *ksr2*^−/−^ mice, it may be beneficial for survival.

GH signaling is defective in the liver but not in the skeletal muscle of *ksr2*^−/−^ mice (Fig. 4), revealing a specific role for KSR2 in regulating hepatic GH signaling in neonates. This observation likely excludes KSR2-dependent regulation of circulating GH, for example, via growth hormone binding protein^48^,^49^. However, the fact that GH signaling is defective in the livers of *ksr2*^−/−^ mice but not in isolated primary *ksr2*^−/−^ hepatocytes indicates a cell non-autonomous action of KSR2 in regulating hepatic GH signaling. Thus, KSR2 is likely to affect GH activation of STAT5 indirectly via a nervous or hormonal mechanism that directly affects hepatic GH signaling. KSR2 is expressed primarily in brain^12^,^17^. It is possible that the lack of KSR2 provides an inappropriate signal, or fails to send a necessary signal, on the metabolic status in the brain to the liver. This raises the possibility that the obesity observed in adult *ksr2*^−/−^ mice is a consequence of KSR2’s action in the brain or a tissue other than adipocytes. Indeed, KSR2 mRNA is barely detectable in white adipose tissue^12^. However, exogenous IGF-1 expression, even though it rescues body length, BMC, and BMD, does not prevent obesity in *ksr2*^−/−^ mice. Thus, the obesity of *ksr2*^−/−^ mice cannot be ascribed to the suppression of IGF-1 expression. The mechanisms through which KSR2 regulates body mass remain unclear. If KSR2 contributes to postnatal programming of adult metabolism, its control over neonatal body mass may be a key contributor. In combination with previous reports^12^,^13^,^19^,^50^,^51^, these data show that KSR2 plays a critical role in organismal growth and metabolism immediately after birth and in adulthood. Defining the physiological mechanisms that mediate KSR2′s action will reveal the extent to which its role in postnatal development contributes to adult energy balance.

## Methods

### Generation and housing of mice

*ksr2*^−/−^ mice were generated by standard techniques targeting exon 4 of the *ksr2* locus^12^. Mice were backcrossed for >10 generations onto the C57BL/6J background as described previously^13^. *fgf21*^−/−^ mice were a gift from Dr. Steven A. Kliewer (UT Southwestern). *fgf21*^−/−^ mice were backcrossed for 7 generations, then crossed with *ksr2*^+/−^ mice. *fgf21*^−/−^ *ksr2*^−/−^ mice were generated from *fgf21*^−/−^ *ksr2*^+/−^ matings. All study protocols were approved by the Institutional Animal Care and Use Committee (University of Nebraska Medical Center, Omaha, NE) and were performed in accordance with federal, state, local, and Association for Assessment and Accreditation of Laboratory Animal Care guidelines. Animals were maintained on a 12 h light/dark schedule with free access to water and laboratory chow (Harlan Teklad 7912).

### Bone mineral content (BMC) and bone mineral density (BMD) measurements

Mice at five weeks of age were anesthetized with isoflurane, nose-to-anus lengths were measured, and the Lunar PIXImus Densitometer (GE Medical Systems) dual energy x-ray absorptiometry (DEXA) was used to measure and calculate total body (exclusive of the head region) BMC and BMD.

### Adenovirus experiments

Ad5RSV-eGFP and Ad5RSV-IGF-1 vectors, expressing EGFP and human IGF-1, respectively, were purchased from the University of Iowa Viral Vector Core Facility. Viral titers were 6 × 10^10^ pfu/ml. Postnatal day 2 (PN2) pups were injected with EGFP-expressing control or IGF-1-expressing virus (4.8 × 10^8^ pfu virus in 20 μl total volume per mouse) once by retro-orbital injection according to the method described previously^29^. Litter sizes of 4–8 were used for the study. Weights were measured every other day from PN4 to PN17.

### Quantitative PCR

Mice were euthanized by CO_2_ inhalation and cervical dislocation at PN17 and older, or by CO_2_ inhalation and decapitation at PN6. Tissues removed from WT and *ksr2*^−/−^ mice were immediately frozen on dry ice or in liquid nitrogen and stored at −80 °C. RNA was extracted using TRI Reagent (Molecular Research Center) and the RNeasy kit (Qiagen). After subsequent treatment with DNase I (Ambion, AM1906), cDNA was synthesized using iScript RT Supermix (BioRad) according the manufacturer’s instructions. Quantitative real-time PCR (qPCR) was performed in a total reaction volume of 20 μl using SsoAdvanced Sybr Green Supermix (BioRad) according to the manufacturer’s instructions. All PCR reactions were performed in duplicate on a Stratagene MxPro3000p detection system, and relative mRNA levels were calculated by the comparative threshold cycle method by using rps18 or 18S rRNA as an internal control. The primer sequences of MUP3 and MUP1/2/6/8 were described previously^52^.

FGF21: 5′-CTACCAAGCATACCCCATCC-3′ and 5′-GCCTACCACTGTTCCATCCT-3′

IGF-1: 5′-TGGATGCTCTTCAGTTCGTG-3′ and 5′-ATCCACAATGCCTGTCTGAG-3′

m+hIGF-1: 5′-AGCAGTCTTCCAACCCAATTA-3′ and 5′-AGGTAGAAGAGATGCGAGGA-3′

18s rRNA: 5′-GTAACCCGTTGAACCCCATT-3′ and 5′- CCATCCAATCGGTAGTAGCG-3′

rps18: 5′-CATGCAGAACCCACGACAGTA-3′ and 5′-CCTCACGCAGCTTGTTGTCTA-3′

IGFALS: 5′-GTACAAGGAACAATGGCTCTGA-3′ and 5′-CTGATGCTCCAGGATCTGTC-3′

MUP3: 5′-AAGAGTGCACCGAAATGACTG3′ and 5′-TGCCAGCCTTTTCTGTTTGTT-3′

MUP1/2/6/8: 5′-GACTTTTTCTGGAGCAAATCCATG-3′ and 5′-GAGCACTCTTCATCTCTTACAG-3′

SOCS1: 5′-CAGAAAAATGAAGCCAGAGACC-3′ and 5′-ATTCCACTCCTACCTCTCCAT-3′

SOCS2: 5′-AATGGTGTGGCAAAGTCTCT-3′ and 5′-CGCCCTCAAGATCCCTT-3′

SOCS3: 5′-TTGTCGGAAGACTGTCAACG-3′ and 5′-GGCTGGATTTTTGTGCTTGT-3′

CIS: 5′-CCGCCCAATTTGCTCCA-3′ and 5′-GCTCCTTTCTCCTTCCATCC-3′

### ELISA

Serum FGF21, GH, and human and mouse IGF-1 levels were measured using a Rat/Mouse FGF21 ELISA kit (Millipore, EZRMFGF21-26K), mouse GH ELISA kit (Millipore, EZRMGH-45K), and mouse IGF-1 ELISA kit (Alpco Immunoassays, 22-IG1MS-E01), respectively. Assays were performed according to the manufacturer’s instructions.

### Western blot

Mice received intraperitoneal injections of 125 μg/kg recombinant human GH in 50 μl PBS. Mice were euthanized 15 minutes after injection. Liver and quadriceps muscle were lysed in lysis buffer containing 25 mM Tris (pH 7.4), 125 mM NaCl, 1 mM MgCl_2_, and 1% NP40 with 2 mM EDTA, 10 mM NaF, 10 μg/ml aprotinin, 20 μM leupeptin, 0.5 mM Na_3_VO_4_, 10 mM β-glycerophosphate, and 1 mM PMSF. Phosphorylation of STAT5b and JAK2 was analyzed via western blot as described previously^12^ with the indicated antibodies. The primary antibodies included STAT5 (Santa Cruz, SC1656), p-STAT5 (Cell Signaling, 9351S), JAK2 (Millipore, 04-001), p-JAK2 (Cell Signaling, 8082S), α tubulin (Santa Cruz, SC23948), and β actin (Santa Cruz, SC47778). Proteins were detected and analyzed using the Odyssey Imaging System (Li-COR).

### Primary hepatocyte culture

Primary hepatocytes were cultured as previously described with the following modifications^53^,^54^. Livers from PN17 neonates were carefully removed and put into Krebs-Ringer HEPES (KRH) buffer (25 mM HEPES, 115 mM NaCl, 5 mM KCl, and 1 mM KH_2_PO_4_) with 5 mM EDTA under continuous gassing (O_2_:CO_2_ = 19:1) and agitated at 100 rpm for 30 min. Livers were rinsed and digested with 40 mg/100 ml collagenase (Sigma, C2139) in KRH buffer with 1 mM CaCl_2_ and agitated at 100 rpm for 1 h. Tissues were triturated to facilitate tissue disaggregation. Suspension buffer (KRH buffer with 0.02 g/ml BSA and 2 mM CaCl_2_) was added and liver digests were filtered through 70-μm filters (Becton Dickinson). Cells were washed sequentially by centrifugation at low speed with suspension buffer, suspension buffer with 45% Percoll, and suspension buffer. The final cell pellets were resuspended in Williams Complete Medium and cell viability was assessed by trypan-blue exclusion. Viable cells were plated at 150,000 cells per well on 24-well plates coated with Collagen IV (Sigma C-7521).

### IGF Binding Protein (IGFBP) Ligand Blotting

IGFBP levels were determined by ligand blotting according to a modified version of the method described previously^55^. Aliquots of mouse serum (0.5 μl each) were run on 13% uniform or 8–18% gradient acrylamide gels under non-reducing conditions and then transblotted to BA85 nitrocellulose. The blots were processed as described previously, then incubated with ~650,000 cpm/ml ^125^I-IGF-2 for 16–20 h at 4 °C. Washed blots were visualized by autoradiography. The major IGFBPs expressed in mouse serum were identified by their electrophoretic mobility based on precedent^23^.

### Statistics

Data are presented as mean ± SEM. Data were analyzed for statistical significance using the unpaired, two-tailed Student’s *t*-test for two group comparisons or ANOVA with the Bonferroni post-hoc test for comparisons with more than two groups (GraphPad Prism). Statistical significance is specified for *p* values less than or equal to 0.05.

## Additional Information

**How to cite this article**: Guo, L. *et al*. Cell non-autonomous regulation of hepatic IGF-1 and neonatal growth by Kinase Suppressor of Ras 2 (KSR2). *Sci. Rep.* **6**, 32093; doi: 10.1038/srep32093 (2016).
